# Uncovering the Expression Pattern of the Costimulatory Receptors ICOS, 4-1BB, and OX-40 in Exhausted Peripheral and Tumor-Infiltrating Natural Killer Cells from Patients with Cervical Cancer

**DOI:** 10.3390/ijms25168775

**Published:** 2024-08-12

**Authors:** Jose Manuel Rojas-Diaz, Fabiola Solorzano-Ibarra, Nadia Tatiana Garcia-Barrientos, Ksenia Klimov-Kravtchenko, Marcela Sofia Guitron-Aviña, Jose Alfonso Cruz-Ramos, Pablo Cesar Ortiz-Lazareno, Pedro Ivan Urciaga-Gutierrez, Miriam Ruth Bueno-Topete, Mariel Garcia-Chagollan, Jesse Haramati, Susana del Toro-Arreola

**Affiliations:** 1 Instituto de Investigación en Enfermedades Crónico Degenerativas, Departamento de Biología Molecular y Genómica, Centro Universitario de Ciencias de la Salud, Universidad de Guadalajara, Sierra Mojada # 950, Colonia Independencia, Guadalajara 44340, Jalisco, Mexico; 2 Laboratorio de Inmunología Traslacional, Departamento de Biología Celular y Molecular, Centro Universitario de Ciencias Biológicas y Agropecuarias, Universidad de Guadalajara, Camino Ramón Padilla Sánchez # 2100, Zapopan 45200, Jalisco, Mexico; 3 Coordinación de Investigación, Subdirección de Desarrollo Institucional, Instituto Jalisciense de Cancerología, Guadalajara 44200, Jalisco, Mexico; 4 Centro de Investigación Biomédica de Occidente, División de Inmunología, Instituto Mexicano del Seguro Social (IMSS), Guadalajara 44340, Jalisco, Mexico; 5 Instituto de Investigación en Ciencias Biomédicas, Departamento de Biología Molecular y Genómica, Centro Universitario de Ciencias de la Salud, Universidad de Guadalajara, Guadalajara 44340, Jalisco, Mexico; 6 Laboratorio de Inmunología, Departamento de Fisiología, Centro Universitario de Ciencias de la Salud, Universidad de Guadalajara, Guadalajara 44340, Jalisco, Mexico

**Keywords:** NK cells, cervical cancer, exhaustion, PD-1, TIGIT, ICOS, 4-1BB, OX-40

## Abstract

Cervical cancer (CC) poses a significant health burden, particularly in low- and middle-income countries. NK cells play a crucial role against CC; however, they can become exhausted and lose their cytotoxic capacity. This work explores the expression of costimulatory receptors (ICOS, 4-1BB, OX-40) in exhausted NK cells from CC patients. Peripheral blood and tumor biopsies were collected, and flow cytometry was used to evaluate the expression of costimulatory receptors in exhausted NK cells. There is an increase of peripheral exhausted NK cells (PD-1^+^TIGIT^+^) in CC patients; this subpopulation has a selectively increased expression of the costimulatory receptors ICOS and 4-1BB. An exhausted population is also highly increased in tumor-infiltrating NK cells, and it shows a dramatically increased expression of the costimulatory receptors ICOS (>15×) and 4-1BB (>10×) compared to peripheral NK cells. The exhausted cells, both in the periphery and in the tumor infiltrating lymphocytes (TILs), are also more likely than non-exhausted NK cell populations (PD-1^−^TIGIT^−^) to express these costimulatory receptors; increases ranging from 2.0× ICOS, 2.4× 4-1BB, and 2.6× OX-40 in CD56^dim^ PBMCs to 1.5× ICOS, 5× 4-1BB, and 10× OX-40 in TILs were found. Our study demonstrates for the first time the increased expression of the costimulatory receptors ICOS, 4-1BB, and OX-40 in peripheral CD56^dim^, CD56^bright^, and tumor-infiltrating NK cells in CC. Targeting these receptors for stimulation could reverse exhaustion and be a promising immunotherapy strategy.

## 1. Introduction

Cervical cancer (CC) continues to be a substantial health problem in low- and middle-income countries, where 90% of cases are diagnosed and the mortality rate is 18 times higher compared to developed countries [1]. In countries like Mexico, CC is the gynecological cancer with the highest mortality rate [2].

The destruction of tumor cells and human papillomavirus-infected cells is mainly carried out by natural killer (NK) cells and cytotoxic T lymphocytes (CTLs). NK cells are a highly heterogeneous population in the tumor microenvironment that plays an essential role in the response to CC [3]. Peripheral NK cells are classically categorized into two groups based on the surface markers that they express: the first group, defined as CD56^bright^ NK cells, are producers of proinflammatory cytokines, such as IFN-γ and TNF-α, whereas the second group, CD56^dim^ NK cells, have higher cytotoxic activity, are the most numerous in circulation, and are associated with a more mature phenotype [4].

NK cells recognize tumor cells through a wide repertoire of inhibitory, activating, and costimulatory receptors, whose balance mediates the signals that determine their activation. Some of the costimulatory receptors that can influence NK cell activation are ICOS (CD278), 4-1BB (CD137), and OX-40 (CD134) [5].

ICOS (also known as CD278) is a glycoprotein of the CD28 family that is expressed as a homodimer on the surface of NK cells and T lymphocytes after activation. The binding of ICOS with ICOSL plays an important role in the homeostasis, development, and function of NK cells, favoring their cytotoxic capacity and IFN-γ production [6,7].

4-1BB (also known as CD137 or TNFRSF9) is a 27 kDa glycoprotein belonging to the TNF receptor superfamily. This inducible receptor is expressed on the surface of NK cells and other hematopoietic cells, such as neutrophils, granulocytes, monocytes, mast cells, eosinophils, and dendritic cells. The binding of 4-1BB with its ligand (4-1BBL) induces the activation of the JNK, ERK, and AKT signaling pathways, which converge in the regulation of the transcription factor NF-κB, and this promotes the antitumor cellular response [8].

OX-40 (also known as CD134 or TNFRSF4) is a transmembrane and inducible 50 kDa glycoprotein belonging to the TNF receptor superfamily. The receptor can be found in activated NK cells and other lymphoid cells. The binding of OX-40 with its natural ligand (OX-40L) can promote NK cell activation, cytokine production, and enhanced NK cytotoxicity against target cells [9].

As a counterpart, NK cells also express inhibitory receptors, such as PD-1 and TIGIT, which have been implicated in exhaustion, a process that may occur in NK cells with chronic exposure to antigens. This exhausted phenotype is characterized not only by the increase of inhibitory receptors but also by a decreased production of effector cytokines, such as IFN-γ and GM-CSF; lower production of cytolytic molecules, such as perforin and granzymes; impaired expression of FasL, TRAIL, and CD107a; dysregulation of proliferation; metabolic dysfunction; and epigenetic reprogramming, as well as reduced cytotoxic activity [10,11,12,13].

We have previously reported an increased expression of the exhausted phenotype (PD-1^+^TIGIT^+^) in peripheral NK cells from women with cervical cancer compared to healthy donors [14]; however, it is still unknown whether these exhausted NK cells can express costimulatory receptors that could be used to revert the exhausted phenotype and improve their cytotoxic capacity.

Thus, this work aims to evaluate the expression of the costimulatory receptors ICOS, 4-1BB, and OX-40 in exhausted peripheral and tumor-infiltrating NK cells from patients with CC.

## 2. Results

### 2.1. Expression of Costimulatory and Inhibitory Receptors in Peripheral NK Cells

**Figure 1:**
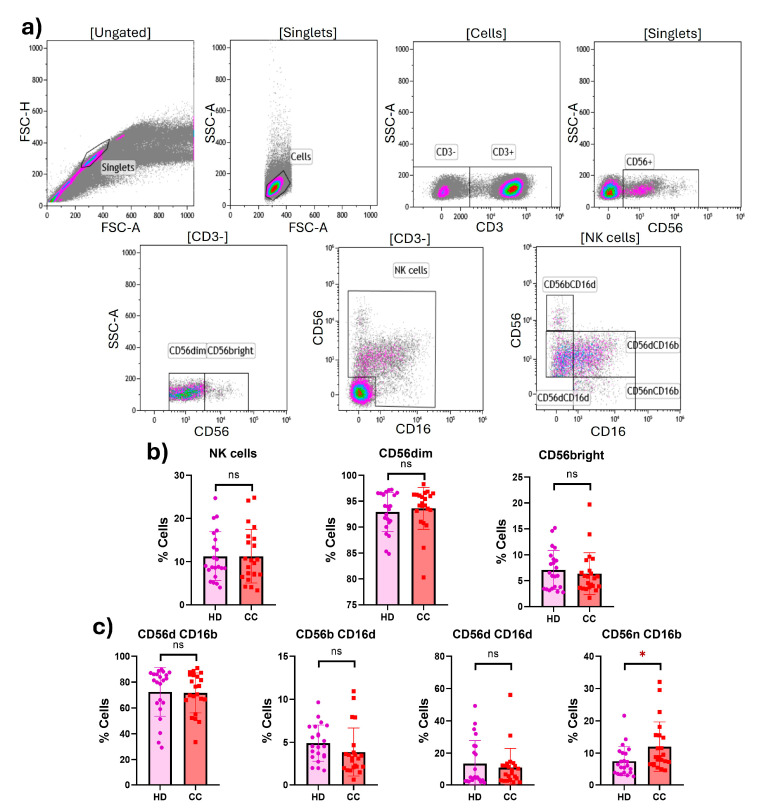
**Percentages of peripheral NK cells from HD and CC patients.** (**a**) Gating strategy for the peripheral NK cell flow cytometry analysis in PBMCs from HD (*n* = 22) and CC patients (*n* = 22). Gated from the lymphocyte region in the singlet population, NK cells were determined as CD3^-^CD56^+^ and classified as CD56^dim^ and CD56^bright^ NK cells. NK cells were further subdivided into four different subsets based on the expression of CD56 and CD16: CD56^dim^CD16^bright^, CD56^bright^CD16^dim^, CD56^dim^CD16^dim^, and CD56^neg^CD16^bright^ NK cells. (**b**) Percentages of total NK cells, CD56^dim^, and CD56^bright^ NK cells in PBMCs from HD and CC patients. (**c**) Percentages of CD56^dim^CD16^bright^, CD56^bright^CD16^dim^, CD56^dim^CD16^dim^, and CD56^neg^CD16^bright^ NK cells in PBMCs from HD and CC patients. *p*-value: * *p* < 0.05; ns: non-significant.

First, we examined the percentages of total NK cells in healthy donors (HD) and cervical cancer (CC) patients (HD: 11.27; CC: 11.27; *p* = 0.9990), as well as the two main populations CD56^dim^ (HD: 92.90; CC: 93.61; *p* = 0.5475) and CD56^bright^ (HD: 7.086; CC: 6.374; *p* = 0.5500) in peripheral blood mononuclear cells (PBMCs) from HD and CC patients; however, no significant statistical differences were found between groups. We further evaluated the four subtypes of NK cells, considering the expression of CD56 and CD16: CD56^dim^CD16^bright^ (HD: 72.47; CC: 71.68; *p* = 0.8791), CD56^bright^CD16^dim^ (HD: 4.912; CC: 3.871; *p* = 0.1719), CD56^dim^CD16^dim^ (HD: 13.48; CC: 10.93; *p* = 0.5278), and CD56^neg^CD16^bright^ (HD: 7.532; CC: 12.00; *p* = 0.0129); while no significant difference was observed in three of the subtypes, the CD56^neg^CD16^bright^ subtype was significantly (although only slightly) increased in CC patients (Figure 1). Additionally, we performed the above analysis comparing NK cell populations from the 11 early-stage samples (I and II FIGO staging) with the 11 late-stage samples (III and IV FIGO staging); no significant differences were found.

**Figure 2:**
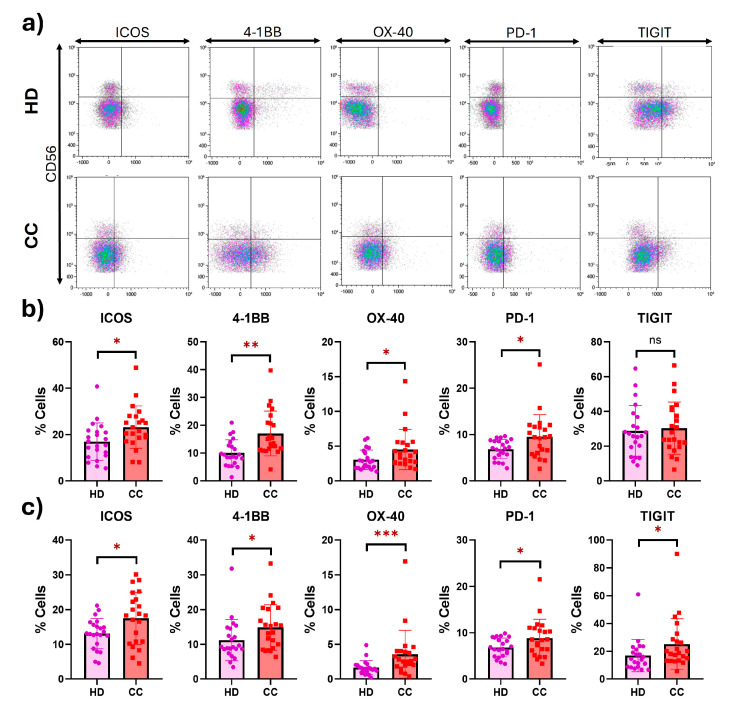
**Expression of costimulatory and inhibitory receptors in peripheral NK cells from HD and CC patients.** (**a**) Representative dot plots of the costimulatory and inhibitory receptors in NK cells from HD and CC patients. (**b**) Receptor expression in peripheral CD56^dim^ NK cells from HD and CC patients. (**c**) Receptor expression in peripheral CD56^bright^ NK cells from HD and CC patients. *p*-value: * *p* < 0.05; ** *p* < 0.01; *** *p* < 0.005; ns: non-significant.

Peripheral CD56^dim^ NK cells from CC patients revealed significantly increased percentages of ICOS (1.4-fold change) (HD: 17.00; CC: 23.25; *p* = 0.0122), 4-1BB (1.7-fold change) (HD: 10.07; CC: 17.08; *p* = 0.0011), OX-40 (1.5-fold change) (HD: 3.034; CC: 4.530; *p* = 0.0352), and PD-1 (1.4-fold change) (HD: 6.859; CC: 9.539; *p* = 0.0200). Similarly, peripheral CD56^bright^ NK cells from CC patients showed significantly increased percentages of ICOS (1.3-fold change) (HD: 13.08; CC: 17.53; *p* = 0.0213), 4-1BB (1.3-fold change) (HD: 11.22; CC: 14.88; *p* = 0.0459), OX-40 (2.2-fold change) (HD: 1.635; CC: 3.579; *p* = 0.0010), PD-1 (1.3-fold change) (HD: 6.832; CC: 8.849; *p* = 0.0443), and TIGIT (1.5-fold change) (HD: 16.92; CC: 25.24; *p* = 0.0322) (Figure 2). MFI values are shown in Supplementary Figure S1. Additionally, we performed an analysis comparing expression levels of ICOS, 4-1BB, OX-40, PD-1, or TIGIT receptors between early- and late-stage CC patients; no significant differences were found.

### 2.2. Peripheral NK Cells with an Exhausted Phenotype Overexpress Costimulatory Receptors

**Figure 3:**
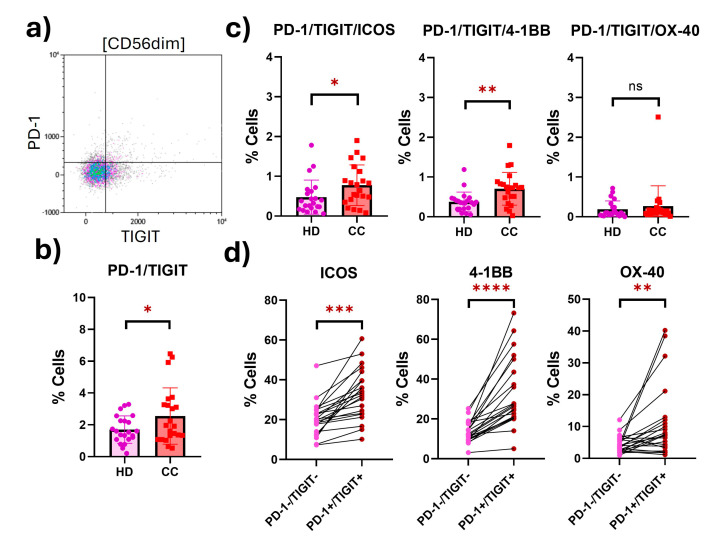
**Expression of costimulatory receptors in peripheral exhausted CD56^dim^ NK cells from HD and CC patients.** (**a**) Representative dot plot of the exhausted population (PD-1^+^TIGIT^+^) CD56^dim^ NK cells from CC patients. (**b**) Putatively exhausted CD56^dim^ NK cell population from HD and CC patients. (**c**) Coexpression of costimulatory receptors in the putatively exhausted CD56^dim^ NK cells. (**d**) Differential coexpression of costimulatory receptors in putatively exhausted (PD-1^+^TIGIT^+^) compared to non-exhausted (PD-1^-^TIGIT^-^) CD56^dim^ NK cells from the same patients. *p*-value: * *p* < 0.05; ** *p* < 0.01; *** *p* < 0.05; **** *p* < 0.01; ns: non-significant.

In peripheral CD56^dim^ NK cells from CC patients, we found a statistically significant increase (1.5-fold) in a putatively exhausted population characterized by the coexpression of the inhibitory receptors PD-1 and TIGIT (HD: 1.702; CC: 2.551; *p* = 0.0500). We evaluated the expression of the costimulatory receptors in this exhausted population, and we found that the putatively exhausted CD56^dim^ NK cells from CC patients significantly overexpressed ICOS (1.6-fold change) (HD: 0.4764; CC: 0.7750; *p* = 0.0500) and 4-1BB (1.9-fold change) (HD: 0.3736; CC: 0.7045; *p* = 0.0024). To verify that the coexpression of costimulatory receptors was differentially expressed in cells with an exhausted phenotype, we evaluated the coexpression of these receptors in putatively exhausted (PD-1^+^TIGIT^+^) against non-exhausted CD56^dim^ NK cells (PD-1^−^TIGIT^−^) from the same patients, and the three costimulatory receptors were shown to be differentially expressed in cells with an exhausted phenotype (Figure 3).

**Figure 4:**
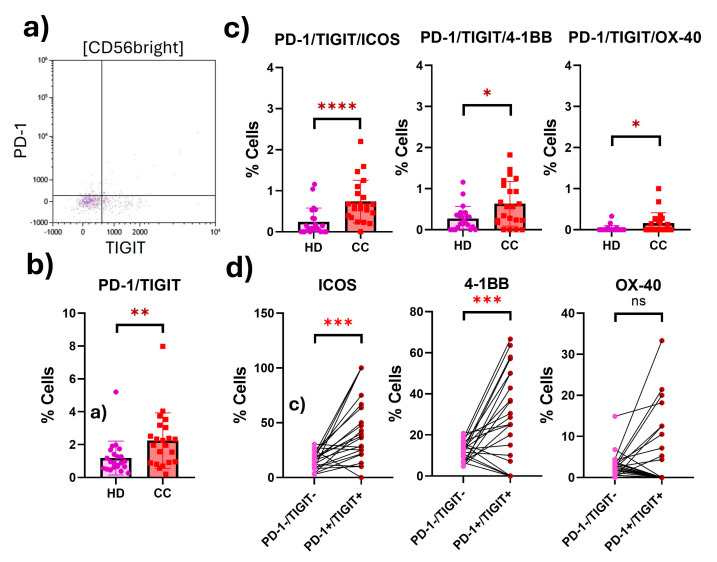
Expression of costimulatory receptors in peripheral exhausted CD56^bright^ NK cells from HD and CC patients. (**a**) Representative dot plot of the exhausted population (PD-1^+^TIGIT^+^) CD56^bright^ NK cells from CC patients. (**b**) Putatively exhausted CD56^bright^ NK cell population from HD and CC patients. (**c**) Coexpression of costimulatory receptors in the putatively exhausted CD56^bright^ NK cells. (**d**) Differential coexpression of costimulatory receptors in putatively exhausted (PD-1^+^TIGIT^+^) compared to non-exhausted (PD-1^−^TIGIT^−^) CD56^bright^ NK cells from the same patients. *p*-value: * *p* < 0.05; ** *p* < 0.01; *** *p* < 0.005; **** *p* < 0.001; ns: non-significant.

We also evaluated the CD56^bright^ NK cells from HD and CC patients, and we found the putatively exhausted population (PD-1^+^TIGIT^+^) significantly increased (1.9-fold) in CC patients compared to HD (HD: 1.184; CC: 2.242; *p* = 0.0045). Putatively exhausted CD56^bright^ NK cells from CC patients overexpressed ICOS (3-fold change) (HD: 0.2432; CC: 0.7373; *p* < 0.0001), 4-1BB (2.8-fold change) (HD: 0.2000; CC: 0.5600; *p* = 0.0287), and OX-40 (6.1-fold change) (HD: 0.02636; CC: 0.1600; *p* = 0.0267) (Figure 4). We found that the expression of both ICOS and 4-1BB was differentially overexpressed in putatively exhausted NK cells (Figure 4).

### 2.3. Tumor-Infiltrating NK Cells with an Exhausted Phenotype Overexpress Costimulatory Receptors

Finally, we evaluated the expression of the costimulatory and inhibitory receptors in tumor-infiltrating NK cells in TILs from eight CC biopsies and compared these results to the expression seen in the peripheral NK cells isolated from CC PBMCs. We found a significant increase (3-fold change) in the costimulatory receptor ICOS (PBMCs: 22.58; TILs: 68.07; *p* < 0.0001) and a 3.9-fold change in the inhibitory receptor PD-1 (PBMCs: 9.230; TILs: 36.19; *p* < 0.0001).

**Figure 5:**
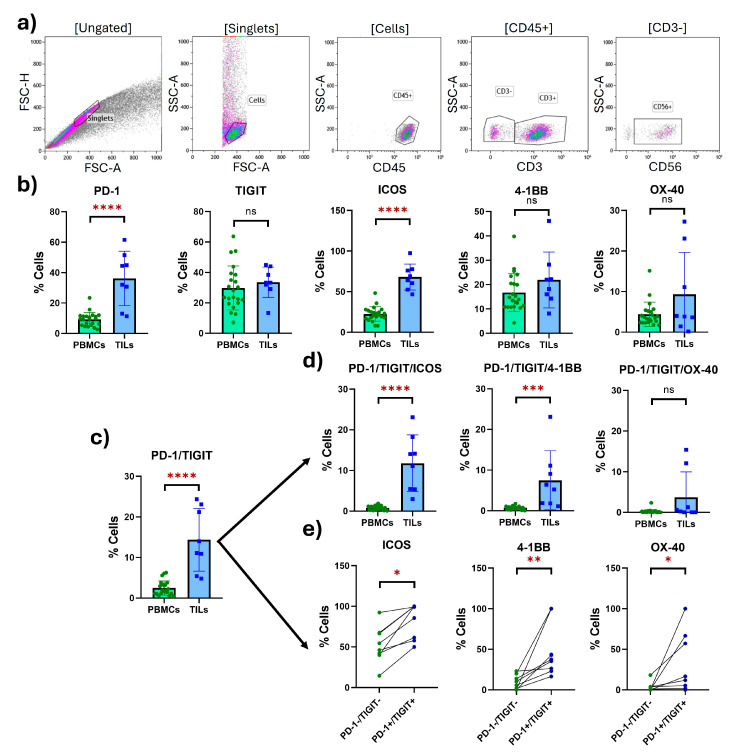
Expression of costimulatory and inhibitory receptors in tumor-infiltrating NK cells. (**a**) Gating strategy for the tumor-infiltrating NK cell flow cytometry analysis in biopsies from CC patients. Gated from the lymphocyte region in the singlet population, NK cells were determined as CD45^+^CD3^−^CD56^+^. (**b**) Receptor expression in peripheral NK cells from PBMCs (*n* = 22) compared to tumor-infiltrating NK cells (*n* = 8) from CC patients. (**c**) Putatively exhausted NK cell population in PBMCs and TILs from CC patients. (**d**) Coexpression of costimulatory receptors in the putatively exhausted NK cells from CC PBMCs compared to TILs. (**e**) Differential coexpression of costimulatory receptors in putatively exhausted (PD-1^+^TIGIT^+^) compared to non-exhausted (PD-1-TIGIT-) tumor-infiltrating NK cells from CC patients. *p*-value: * *p* < 0.05; ** *p* < 0.01; *** *p* < 0.005; **** *p* < 0.001; ns: non-significant.

The putatively exhausted tumor-infiltrating NK cells (PD-1^+^TIGIT^+^) were significantly increased (5.8-fold change) compared to peripheral NK cells from CC patients. The exhausted population in the tumor dramatically overexpressed ICOS (15.4-fold change) compared to peripheral NK cells (PBMCs: 0.7673; TILs: 11.79; *p* < 0.0001), while 4-1BB was overexpressed 10.6-fold in TILs compared to PBMCs (PBMCs: 0.7073; TILs: 7.474; *p* = 0.0001) (Figure 5). For easier reference, a summary of all the data is presented in tabular format in Supplementary Table S1.

## 3. Discussion

Here, we report a significant increase in PD-1 expression in peripheral CD56^dim^ and CD56^bright^ NK cells among CC patients compared to HD. PD-1 overexpression has been reported in peripheral CD56^dim^ and CD56^bright^ NK cells from patients with digestive cancers, such as esophageal, liver, colorectal, gastric, and biliary cancer, which correlates with poorer survival outcomes [15]. Similarly, PD-1 was observed in peripheral CD56^dim^ NK cells from renal cell carcinoma patients and exhibited rapid reduction following surgical tumor resection [16]. Intriguingly, we found that tumor-infiltrating NK cells displayed PD-1 levels nearly four times higher than peripheral cells, consistent with findings by Liu et al. in digestive cancers [15].

We observed minimal expression of PD-1 in HD; nevertheless, it has been reported that although PD-1 surface expression is absent or low in NK cells from healthy individuals, a cytoplasmic reservoir of PD-1 mRNA and protein is present in all resting NK cells. The existence of this intracellular pool implies that PD-1 can swiftly be expressed on the cell surface membrane, thereby inhibiting NK cell activation in response to various stimuli [17].

Increased expression of PD-1 is considered a pivotal marker of exhaustion in both T and NK cells. Not only does PD-1 serve as a hallmark of exhausted NK cells, but its signaling plays a significant role in driving the exhaustion process [18]. PD-1^+^ NK cells show poor cytokine-induced proliferation and lower degranulation and cytokine production compared with PD-1^−^ cells [19]. In patients with Kaposi sarcoma, PD-1 is upregulated on CD56^dim^CD16^+^ NK cells, and it contributes to dysfunction rather than merely serving as a marker of dysfunctional NK cells. PD-1^+^ NK cells exhibit hyporesponsiveness ex vivo when directly triggered via activating receptors, leading to their failure to degranulate and release IFN-γ [20]. Therefore, our results regarding the overexpression of PD-1 in peripheral and tumor-infiltrating NK cells from CC patients could suggest a state of hyporesponsiveness associated with exhaustion.

Furthermore, we found an increased percentage of expression of the inhibitory receptor TIGIT in peripheral CD56^bright^ NK cells from CC patients compared to HD. TIGIT has been linked to NK cell exhaustion in some cancers, such as colon cancer [21]. The high expression levels of TIGIT have been associated with impaired NK and T cell function, resulting in significantly reduced secretion of activation factors, such as CD107a, IFN-γ, and TNF-α, leading to the promotion of immune evasion [22]. TIGIT exerts its inhibitory effects on NK cells by inhibiting NF-κB activity and IFN-γ production while also competing with the costimulatory receptor DNAM-1 [23] and inducing IL-10 expression by dendritic cells [24]. Hence, an increased expression of TIGIT could have a strong inhibitory effect on NK cells, similar to PD-1.

Interestingly, we reported putatively exhausted peripheral CD56^dim^ and CD56^bright^ NK cell populations in CC patients, characterized by the coexpression of PD-1 and TIGIT, which stand out as primary inhibitory receptors in both T and NK cells. Moreover, this exhausted population was notably enriched in tumor-infiltrating NK cells. In certain cancers, such as colorectal cancer, the upregulation of these receptors has been linked to disease progression [25], while in lung squamous cell carcinoma, a strong correlation exists between PD-1 and TIGIT densities, and the coexpression of their ligands holds promise as both a prognostic factor in the disease and a potential predictive biomarker for dual-targeting immunotherapy [26]. Additionally, strong coexpression between both inhibitory receptors has been observed in tumor antigen-specific CD8^+^ T cells in the periphery and at tumor sites in patients with advanced melanoma [27], while in lung adenocarcinoma, this coexpression implies the existence of suppressive phenotypes of tumor-infiltrating lymphocytes, which is closely correlated with poor clinical outcomes [28].

In most studies, PD-1 and TIGIT are typically assessed separately within cell populations. However, in acute myeloid leukemia, a unique CD8^+^ T cell subset that expressed PD-1 and TIGIT but displayed lower levels of DNAM-1 has been linked to failure to achieve remission after induction chemotherapy. These PD-1^+^TIGIT^+^ T cells are dysfunctionally characterized by the reduced expression of intracellular IFN-γ and TNF-α [29]. Similarly, in B-cell chronic lymphocytic leukemia, Farhat et al. found an NK cell phenotype involving the coexpression of the potent inhibitory checkpoints PD-1 and TIGIT, which was not observed in NK cells from HD but constituted over 2% of the NK cells in many patients. Furthermore, they observed that PD-1^+^ NK cells express lower levels of activating receptors (NKG2D, NKp30, NKp46, and DNAM-1) and exhibit reduced functionality, which could be partially reversed with an anti-PD-L1 blockade [30].

The functional exhaustion of NK cells in tumors is occasionally accompanied by the downregulated expression of certain activating receptors on NK cells, such as NKG2D, natural cytotoxicity receptor (NCR) family (NKp30, NKp44, and NKp46), DNAM-1, and 2B4 [10]. However, limited research has focused on certain costimulatory receptors in NK cells that have historically been more extensively studied in T cells, such as the receptors ICOS, 4-1BB, and OX-40.

We found an increased expression of the costimulatory receptor ICOS in peripheral CD56^dim^ and CD56^bright^ NK cells in CC patients, as well as a 3-fold increase in expression in tumor-infiltrating NK cells. Furthermore, in a study showing the importance of NK cell dysfunction in multiple myeloma, ICOS expression levels were found to be higher in bone marrow CD56^bright^ NK cells from relapsed/refractory patients, while TIGIT and TIM3 were increased [31].

Traditionally, ICOS exhibits a dualistic role in tumor immunity. On the one hand, it fosters the activation of antitumor cytotoxic T cells; however, on the other hand, it enhances Treg immunosuppressive activity in the tumor microenvironment. As a result, the role and prognostic significance of ICOS may be different depending on the type of cancer [32], underscoring the importance of ongoing critical inquiry into its function. This inquiry remains crucial since ICOS mRNA overexpression in tumor biopsies compared to normal tissues has been detected in more than 30 types of cancers [33].

Among the many antitumor effects of ICOS reported, it has been observed that higher levels improve patient prognosis in lung adenocarcinoma and are associated with the inhibition of tumor progression and a healthier tumor immune microenvironment (enriched T cell numbers and improved ImmuneScore and ESTIMATEscore) [34]. Additionally, a higher percentage of ICOS^+^ T cells in peripheral blood may serve as a marker for good prognosis in colorectal cancer [35], whereas lower ICOS expression is linked to a poorer prognosis in oral squamous cell carcinoma [36]. Moreover, ICOS may mediate the stimulation of antitumor immunity, and its expression is a survival predictor in head and neck squamous cell carcinoma [37]. Collectively, these data underscore the potential of ICOS as a pertinent target for cancer immunotherapies.

We also found an increased expression of the costimulatory receptor 4-1BB in peripheral CD56^dim^ and CD56^bright^ NK cells in CC patients compared to HD. This pattern has also been seen in chronic lymphocytic leukemia, where peripheral NK cells express a significant increase in 4-1BB compared to HD [38]. Likewise, 4-1BB can be upregulated in NK cells after receiving monoclonal antibody therapy, such as Trastuzumab (anti-HER2), Cetuximab (anti-EGFR), and Rituximab (anti-CD20) [39]. Recently, in cervical cancer, it has been reported that increased coexpression of 4-1BB with PD-1 in CD8^+^ TILs is associated with improved prognosis and immunotherapy response [40].

In melanoma, TILs exhibit an increased expression of 4-1BB that is notably coexpressed with PD-1 in CD8^+^ T cells. Given the prevalence of PD-1^+^4-1BB^+^ TILs, targeting them in cancer immunotherapy appears reasonable despite the potential exhaustion [41], especially since it has been demonstrated that 4-1BB can trigger human NK cell proliferation and activation [42] and could restore cytotoxic activity in suppressed NK cells [43]. These findings underscore the potential of 4-1BB as a valuable target for NK cell-based immunotherapies.

Similarly, we found an increased expression of the costimulatory receptor OX-40 in peripheral CD56^dim^ and CD56^bright^ NK cells in CC patients. Although OX-40 expression and its role in NK cells have been minimally investigated, it has been detected in CD4^+^ TILs in breast cancer, sarcoma, melanoma, and colorectal cancer [44]. Moreover, high OX-40 expression in tumor infiltrates has previously been found in non-small cell lung cancer, and it has been associated with a better prognosis [45]. Additionally, it has been found that OX-40 expression is elevated in hepatocellular carcinoma compared to adjacent liver tissue, and its expression correlates with PD-1 and other exhaustion markers [46].

### Limitations and Strengths of the Study

It is important to note that we have focused this study on what we termed putatively exhausted NK cells. While it is well established that the expression of two or more inhibitory checkpoint molecules is a cardinal feature of exhausted T cells [47], it is not as clear in NK cells; indeed, there is some discussion over the role of PD-1 in NK cells [48]. We cannot be certain that the NK cell populations that we describe have encountered their ligands PD-L1 and CD155 and are effectively exhausted. Further studies to clarify the exhaustion status (loss of cytotoxicity, decrease in IFN-γ production, decrease in perforin liberation, etc.) of these PD-1^+^TIGIT^+^ cell populations will be necessary. Additionally, our study size did not allow us to find a statistically significant difference between CC staging; with a larger study size, it is possible that these cell populations might be more notably expressed at different time points during the progression of the disease. While our results with respect to the increase in TILs versus PBMCs are striking, it is important to note that, to a certain extent, this is expected; one expects to see more exhaustion and more immune cells in the tumor than in the periphery. As we are the first group to evaluate these co-stimulatory receptors in CC TILs, we are not able to verify if our results are concordant with the literature. It is not clear what will be more clinically relevant: the increase in these populations in CC patients versus controls, the increase in these populations in patient TILs versus patient PBMCs, or the increase in these populations in double-positive versus double-negative PD1 TIGIT TILs. Strengths of the study include the following: we report for the first time that peripheral CD56^dim^ and CD56^bright^ NK cells exhibiting a putatively exhausted phenotype (PD-1^+^TIGIT^+^) express costimulatory receptors at differentially increased levels in CC patients compared to HD. Remarkably, within the putatively exhausted tumor-infiltrating NK cell population, the coexpression of ICOS was increased by more than 15-fold compared to the same population in peripheral NK cells from CC patients, while the expression of 4-1BB was more than 10-fold increased. When we analyzed matched PD-1^+^TIGIT^+^ cells versus the non-exhausted NK cells (PD-1^−^TIGIT^−^) from the same patients, it is clear that there exists a significant increase in activating receptors, both in PBMCs and TILs; perhaps a mechanism to reinvigorate these cells, apart from the blockade of inhibitory receptors, could be explored. Taken together, our findings reveal insights into the immune landscape of cervical cancer, emphasizing the increased expression of costimulatory receptors in exhausted NK cell subsets, which might be exploited for the design of novel therapies focused on the agonistic stimulation of exhausted NK cells in CC patients.

## 4. Materials and Methods

### 4.1. Participants and Sample Collection

Women with recently diagnosed cervical cancer (CC) without previous treatment from the Antiguo Hospital Civil de Guadalajara “Fray Antonio Alcalde” and the OPD Instituto Jalisciense de Cancerología were enrolled in this study. Clinically healthy donors (HD) participated as the control group.

Participants included 22 CC patients naïve to treatment (11 patients in early stages I and II and 11 patients in late stages III and IV, according to FIGO staging) with a mean age of 40 years old (range: 25–73), as well as 22 age-matched HD with a mean age of 39 years old (range: 25–72). Peripheral blood samples were collected in EDTA-coated tubes (Becton Dickinson, Franklin Lakes, New Jersey, USA; cat: 366643), and peripheral blood mononuclear cells (PBMCs) were isolated through density gradient centrifugation using Lymphoprep (Stemcell Technologies, Vancouver, British Columbia, Canada; cat: 07851).

Tumor biopsies were obtained from eight patients in stages II and III, and tumor-infiltrating lymphocytes (TILs) were subsequently isolated by enzymatic dissociation using enzyme H (Milteny Biotec, Bergisch Gladbach, Germany; cat: 130-095-929) and enzyme A (Miltenyi Biotec, Bergisch Gladbach, Germany; cat: 130-095-929) following the protocol of Okolo et al. [49].

PBMCs and TILs were stored frozen in liquid nitrogen at a temperature of −196 °C until cytometry analysis. Thawed cell viability was determined by trypan blue dye exclusion, and cells were stained only when viability was greater than or equal to 90%.

### 4.2. Flow Cytometry

Multiparametric flow cytometry was used to analyze the expression of inhibitory (PD-1 and TIGIT) and costimulatory receptors (ICOS, 4-1BB, and OX-40) in NK cells (CD3^-^CD56^+^). In the case of TILs, a CD45 antibody was added to the cocktail to discriminate immune cells from other cells forming the tumor. Viability was evaluated in every sample in an independent staining using Zombie NIR™, and every sample showed a viability higher than 90%. The following antibodies were used to stain 5 × 10^5^ cells: CD3-FITC (fluorescein isothiocyanate) (Biolegend, San Diego, CA, USA; cat: 300406); CD56-PE/Cy7 (phycoerythrin/cyanine 7) (Biolegend, San Diego, CA, USA; cat: 362510); CD16-PerCP/Cy5.5 (peridinin chlorophyll/cyanine 5.·5) (Biolegend, San Diego, CA, USA; cat: 302028); CD45-AF700 (Alexa Fluor 700) (Biolegend, San Diego, CA, USA; cat: 304024); ICOS-APC (allophycocyanin) (Biolegend, San Diego, CA, USA cat: 313510); 4-1BB-PE (phycoerythrin) (Biolegend, San Diego, CA, USA; cat: 309804); OX-40-APC/Fire750 (allophycocyanin/fire 750) (Biolegend, San Diego, CA, USA; cat: 350032); PD-1-BV421 (brilliant violet 421) (Biolegend, San Diego, CA, USA; cat: 329920); TIGIT-BV510 (brilliant violet 510) (Biolegend, San Diego, CA, USA; cat: 372738); and Zombie NIR Fixable Viability Kit (Biolegend, San Diego, CA, USA cat: 423105).

Data acquisition was performed using the Life Technologies Attune NxT Acoustic Focusing Cytometer. Compensation was made with compensation beads (Becton Dickinson, Franklin Lakes, NJ, USA; cat: 55284), and 250,000 events from the lymphocyte gate were acquired from every sample.

Single and coexpression of costimulatory and inhibitory receptors were analyzed in peripheral and tumor-infiltrating NK cell populations using Kaluza software version 2.1 (Beckman Coulter, San Jose, CA, USA) following the gating strategies outlined by Solorzano et al. and Del Zotto et al. [14,50].

### 4.3. Statistical Analysis

Normality was tested using the D’Angostino–Pearson normality test. When the data were normally distributed, an unpaired *t*-test was performed, whereas in the nonparametric data, a Mann–Whitney test was performed. *p*-values < 0.05 were considered statistically significant. GraphPad Prism V10 was used for statistical analysis.

## 5. Conclusions

Immune exhaustion stands as a pivotal factor in shaping the antitumor response. While exhausted NK cells are characterized by the increased expression of inhibitory receptors, our study marks a significant milestone by uncovering for the first time the increased expression of the costimulatory receptors ICOS, 4-1BB, and OX-40 in peripheral CD56^dim^, CD56^bright^ NK cells, as well as in tumor-infiltrating NK cells. These findings are particularly noteworthy within phenotypically exhausted cells, presenting a promising avenue for immunostimulation aimed at reversing exhaustion and enhancing the response against cervical cancer cells.

## Data Availability

Data are available upon request.

## Supplementary Materials

The following supporting information can be downloaded at: https://www.mdpi.com/article/10.3390/ijms25168775/s1.
