# Supplementary material for: Uncovering the Expression Pattern of the Costimulatory Receptors ICOS, 4-1BB, and OX-40 in Exhausted Peripheral and Tumor-Infiltrating Natural Killer Cells from Patients with Cervical Cancer
Source: Int J Mol Sci. 2024 Aug 12;25(16):8775. doi: 10.3390/ijms25168775 (PMC11354483; doi:10.3390/ijms25168775)
Supplement: Supplementary file 1 [file ijms-25-08775-s001.zip › ijms-3114592-supplementary.pdf]

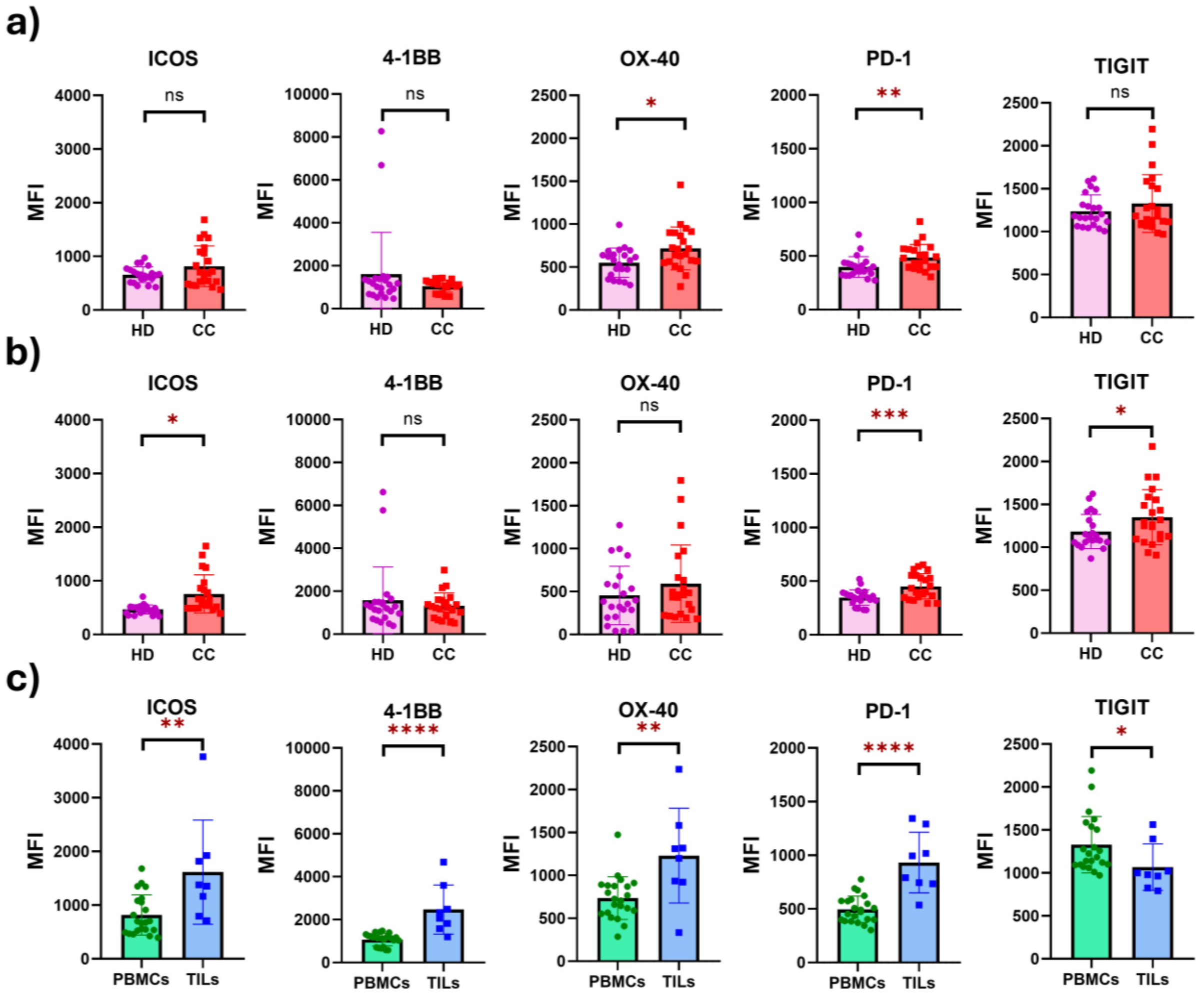


**Figure S1. Mean Fluorescence Intensity (MFI) of costimulatory and inhibitory receptors in NK cells from HD and CC patients. a)** MFI of receptors in peripheral CD56$^{dim}$ NK cells from HD and CC patients. **b)** MFI of receptors in peripheral CD56$^{bright}$ NK cells from HD and CC patients. **c)** MFI of receptors in tumor-infiltrating NK cells versus peripheral NK cells from CC patients.

**Table S2: Data Summary**

| **CD56$^{dim}$ NK cells** | % | | | | MFI | | | |
|---|---|---|---|---|---|---|---|---|
| | HD | CC | FC | *p* | HD | CC | FC | *p* |
| ICOS | 17.00 | 23.25 | 1.4X | 0.0122[2] | 658.6 | 815.8 | - | 0.0762[1] |
| 4-1BB | 10.07 | 17.08 | 1.7X | 0.0011[1] | 1655 | 1055 | - | 0.7715[2] |
| OX-40 | 3.034 | 4.530 | 1.5X | 0.0352[1] | 551.5 | 717.5 | 1.3X | 0.0139[1] |
| PD-1 | 6.859 | 9.539 | 1.4X | 0.0200[1] | 397.5 | 486.5 | 1.2X | 0.0068[2] |
| TIGIT | 28.83 | 30.39 | - | 0.7281[1] | 1239 | 1327 | - | 0.2925[1] |
| **CD56$^{bright}$ NK cells** | % | | | | MFI | | | |
| | HD | CC | FC | *p* | HD | CC | FC | *p* |
| ICOS | 13.08 | 17.53 | 1.3X | 0.0213[1] | 466 | 755.7 | 1.6X | 0.0006[1] |
| 4-1BB | 11.22 | 14.88 | 1.3X | 0.0459[2] | 1579 | 1320 | - | 0.7187[2] |
| OX-40 | 1.635 | 3.579 | 2.2X | 0.0010[2] | 454.4 | 592 | - | 0.2593[1] |
| PD-1 | 6.832 | 8.849 | 1.3X | 0.0443[1] | 347.2 | 451.4 | 1.3X | 0.0010[1] |
| TIGIT | 16.92 | 25.24 | 1.5X | 0.0322[2] | 1183 | 1352 | 1.1X | 0.0421[1] |
| **Tumor-infiltrating NK cells** | % | | | | MFI | | | |
| | PBMCs | TILs | FC | *p* | PBMCs | TILs | FC | *p* |
| ICOS | 22.58 | 68.07 | 3X | <0.0001[1] | 815.4 | 1614 | 2X | 0.0025[1] |
| 4-1BB | 16.75 | 21.91 | - | 0.2015[1] | 1067 | 2469 | 2.3X | <0.0001[1] |
| OX-40 | 4.420 | 9.324 | - | 0.5651[2] | 735.3 | 1230 | 2.5X | 0.0052[2] |
| PD-1 | 9.230 | 36.19 | 3.9X | <0.0001[2] | 495.5 | 931.6 | 1.9X | <0.0001[1] |
| TIGIT | 29.78 | 33.54 | - | 0.5039[1] | 1328 | 1068 | 0.8X | 0.0134[1] |

| **CD56$^{dim}$ NK cells** | % | | | | % | | | |
|---|---|---|---|---|---|---|---|---|
| | HD | CC | FC | *p* | CC PD-1-TIGIT- | CC PD-1+TIGIT+ | FC | *p* |
| PD-1/TIGIT | 1.702 | 2.551 | 1.5X | 0.0500[1] | | | | |
| PD-1/TIGIT/ICOS | 0.4764 | 0.7750 | 1.6X | 0.0302[2] | 20.30 | 32.97 | 2X | 0.0004[2] |
| PD-1/TIGIT/4-1BB | 0.3736 | 0.7045 | 1.9X | 0.0024[2] | 13.44 | 32.57 | 2.4X | <0.0001[1] |
| PD-1/TIGIT/OX-40 | 0.1882 | 0.2655 | - | 0.4111[2] | 4.325 | 11.25 | 2.6X | 0.0069[2] |
| **CD56$^{bright}$ NK cells** | % | | | | % | | | |
| | HD | CC | FC | *p* | CC PD-1-TIGIT- | CC PD-1+TIGIT+ | FC | *p* |
| PD-1/TIGIT | 1.184 | 2.242 | 1.9X | 0.0045[2] | | | | |
| PD-1/TIGIT/ICOS | 0.2432 | 0.7373 | 3X | <0.0001[2] | 15.47 | 42.77 | 2.8X | 0.0002[1] |
| PD-1/TIGIT/4-1BB | 0.2750 | 0.6336 | 2.3X | 0.0287[2] | 12.14 | 29.43 | 2.5X | 0.0008[1] |
| PD-1/TIGIT/OX-40 | 0.02636 | 0.1600 | 6X | 0.0267[2] | 3.094 | 6.601 | - | 0.5593[2] |
| **Tumor-infiltrating NK cells** | % | | | | % | | | |
| | PBMCs | TILs | FC | *p* | TILs PD-1-TIGIT- | TILs PD-1+TIGIT+ | FC | *p* |
| PD-1/TIGIT | 2.485 | 14.36 | 5.8X | <0.0001[1] | | | | |
| PD-1/TIGIT/ICOS | 0.7673 | 11.79 | 15.4X | <0.0001[1] | 53.03 | 81.79 | 1.5 | 0.0227[1] |
| PD-1/TIGIT/4-1BB | 0.7073 | 7.474 | 10.6X | 0.0001[1] | 9.344 | 47.26 | 5X | 0.0048[1] |
| PD-1/TIGIT/OX-40 | 0.2509 | 3.706 | - | 0.4253[2] | 3.364 | 32.40 | 10X | 0.0348[2] |

[1] Unpaired *t* test

[2] Mann-Whitney U test
